# Chitosan Coated Textiles May Improve Atopic Dermatitis Severity by Modulating Skin Staphylococcal Profile: A Randomized Controlled Trial

**DOI:** 10.1371/journal.pone.0142844

**Published:** 2015-11-30

**Authors:** Cristina Lopes, Jose Soares, Freni Tavaria, Ana Duarte, Osvaldo Correia, Oksana Sokhatska, Milton Severo, Diana Silva, Manuela Pintado, Luis Delgado, Andre Moreira

**Affiliations:** 1 Laboratory of Immunology, Basic and Clinical Immunology Unit, Faculty of Medicine, University of Porto, Porto, Portugal; 2 Immunoallergology Unit, Hospital Pedro Hispano, Matosinhos, Portugal; 3 CBQF–Centro de Biotecnologia e Química Fina–Laboratório Associado, Escola Superior de Biotecnologia, Universidade Católica Portuguesa, Porto, Portugal; 4 Dermatology Center Epidermis, Instituto CUF, Porto, Portugal; 5 Department of Medical Education and Simulation, Faculty of Medicine, University of Porto, Porto, Portugal; 6 Department of Epidemiology, Predictive Medicine and Public Health, Faculty of Medicine, University of Porto, Porto, Portugal; 7 Immunoallergology Department, Centro Hospitalar São João, Porto, Portugal; Glaxo Smith Kline, DENMARK

## Abstract

**Background:**

Atopic dermatitis (AD) patients may benefit from using textiles coated with skin microbiome–modulating compounds. Chitosan, a natural biopolymer with immunomodulatory and antimicrobial properties, has been considered potentially useful.

**Objective:**

This randomized controlled trial assessed the clinical utility of chitosan-coated garment use in AD.

**Methods:**

Of the 102 patients screened, 78 adult and adolescents were randomly allocated to overnight use of chitosan-coated or uncoated cotton long-sleeved pyjama tops and pants for 8 weeks. The primary outcome was change in disease severity assessed by Scoring Atopic dermatitis index (SCORAD). Other outcomes were changes in quality of life, pruritus and sleep loss, days with need for rescue medication, number of flares and controlled weeks, and adverse events. Changes in total staphylococci and *Staphylococcus aureus* skin counts were also assessed. Comparisons were made using analysis of variance supplemented by repeated measures analysis for the primary outcome. Interaction term between time and intervention was used to compare time trends between groups.

**Results:**

Chitosan group improved SCORAD from baseline in 43.8%, (95%CI: 30.9 to 55.9), *P* = 0.01, placebo group in 16.5% (-21.6 to 54.6); *P* = 0.02 with no significant differences between groups; Dermatology Quality of life Index Score significantly improved in chitosan group (*P* = 0.02) and a significant increase of skin Coagulase negative Staphylococci (*P* = 0.02) was seen.

**Conclusions:**

Chitosan coated textiles may impact on disease severity by modulating skin staphylococcal profile. Moreover, a potential effect in quality of life may be considered.

**Trial Registration:**

ClinicalTrials.gov NCT01597817

## Introduction

Atopic dermatitis (AD) is a chronic, relapsing inflammatory skin disease with a considerable social and economic burden. In industrialized countries, it has an estimated prevalence of up to 20% in children and 2% in adults [1]. Its pathophysiology is complex and involves skin barrier defects, immunological deregulation, and genetic predisposition [2]. These changes frequently lead to skin colonization with *Staphylococcus aureus*, which is able to produce virulence factors that perpetuate inflammation, even in normal-appearing skin [3]. Disease management demands an integrated approach, aimed not only at controlling skin inflammation and ensuring hydration, but also at regulating the skin microbiome [4–6].

While several recent studies have reported the utility of functional textiles with antimicrobial and antipruritic properties in AD [7, 8], a recent systematic review and meta-analysis by our group found that the recommendation for its use was weak due to the low quality of supporting evidence [9]. These results underscored the need for studies with improved methodology and new compounds. Chitosan, a biopolymer [10], has been considered a promising candidate for use in AD due to its with repair and antiseptic properties [11–13]. Chitosan-coated fabrics with proven inhibitory activity against *S*. *aureus* were considered potentially useful in AD management, but their clinical utility on a real life setting has never been studied.

This randomized controlled clinical trial assessed the clinical utility of chitosan-coated garments in AD patients.

## Methods

**Fig 1:**
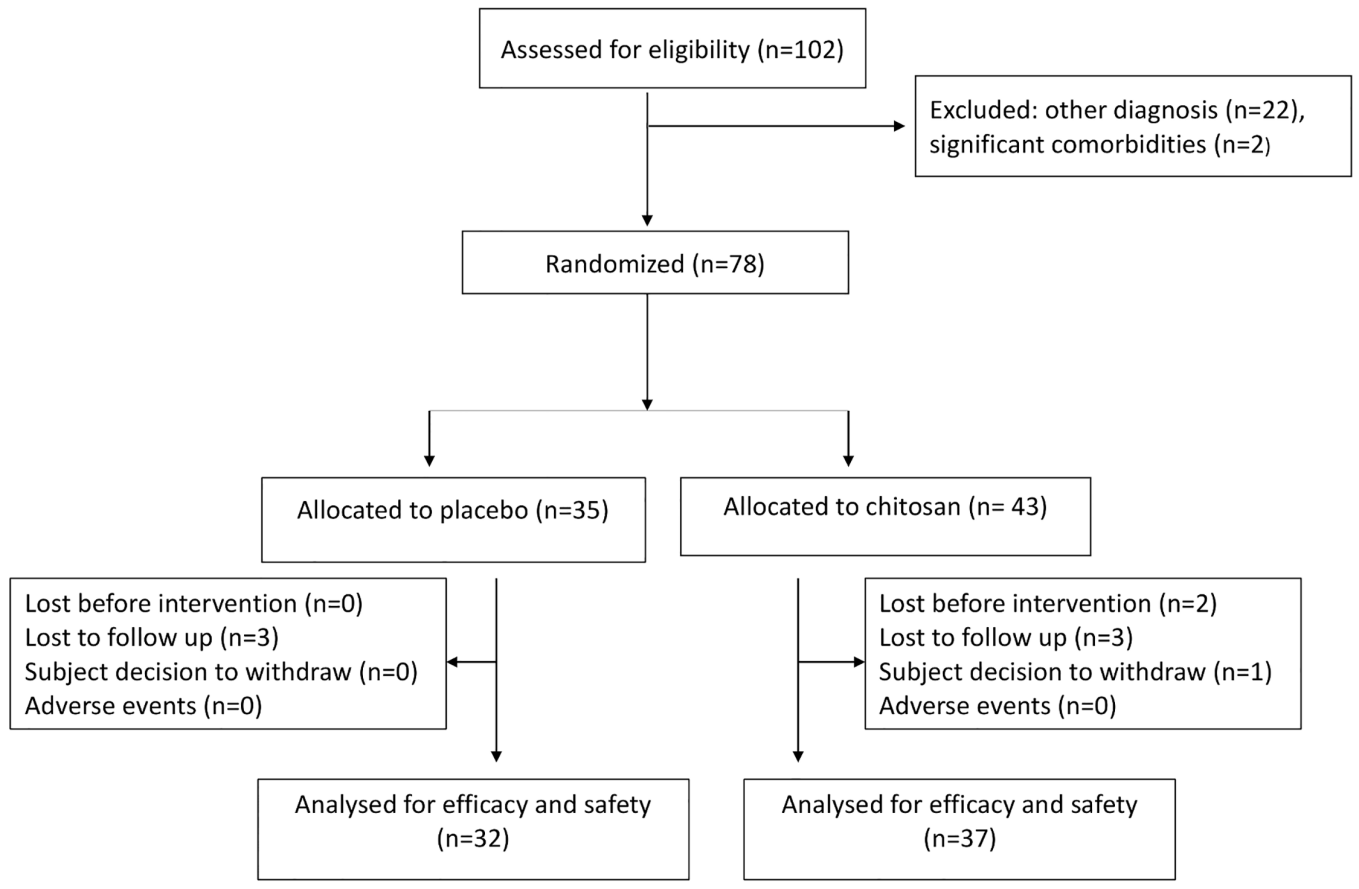
Flow chart of participants through the study.

This is a randomized, double-blind, placebo-controlled, single-center trial. Fig 1 shows the flow of participants. Trial registrations: ClinicalTrials.gov Identifier: NCT01597817. Protocol Registration and Results System account administration delay in releasing the record due to informatics issues caused that the trial was registered after enrolment of participants had started. The authors confirm that all ongoing and related trials for this intervention are registered. Ethics committee approved the study at 6^th^ September 2011, patients recruitment and follow up occurred between December 2011 and June 2012.

### Recruitment

Subjects were invited to participate in the trial during hospital visits, through trial posters on bulletin boards in hospitals, newspaper and Internet advertisements.

### Inclusion and exclusion criteria

Subjects older than 12 years with a diagnosis of AD [14] were eligible for participation following provision of written informed consent. Excluded were patients with severe skin disease other than AD (e.g., psoriasis); secondary infections; major systemic diseases; women who were pregnant and subjects unable to comply with study and follow-up procedures.

Patients who met any of the following criteria were withdrawn from the study: use of topical or systemic antibiotics during the study; withdrawal of consent; detection of significant protocol violations; and investigator’s decision to withdraw the patient due to adverse effects such as skin infections.

### Sample size

Sample size calculations were performed to determine the number of participants needed to detect effect sizes based on minimal clinically important differences in the SCORAD index. Results showed that 42 patients were needed in this two-treatment parallel-design study to detect a treatment difference with a two-sided 0.05 significance level and a probability of 81% if the true difference in SCORAD between interventions was 8.7 units [15].

### Randomization, allocation and blinding

Subjects were randomly assigned to one of two interventions through computer-generated random numbers. The randomization was performed by an independent researcher;the randomization table and intervention codes were kept by the independent researcher in an opaque sealed envelope up to completion of data analysis. A study nurse established phone contact with the independent researcher, who informed the nurse which treatment package was to be assigned to which patient.

A hundred and two patients were assessed for eligibility; twenty-four were excluded because they did not meet inclusion criteria: 22 because the medical diagnosis of AD was not confirmed by the investigation team and two because of significant comorbidities (multiple sclerosis and diabetes mellitus type 1). Seventy-eight were randomized: thirty five to placebo and forty three to chitosan groups. In chitosan group two patients were lost before receiving the intervention and one patient decided to withdraw because of disease progression. In both groups three patients were lost to follow up due to impossibility to attain medical visits (Fig1).

### Treatment protocol and intervention

The study consisted of a 2-week run-in period and an intervention period of 8 weeks (S1 Table). Eligibility to participate was determined at the screening visit. At the end of the run-in period, the patients were examined by the same physician as in the first visit and those with a change in SCORAD of below 10% with respect to baseline were considered eligible for randomization. Participants were randomized to receive either an uncoated pair of cotton pyjamas or a pair of cotton pyjamas coated with chitosan (ChitoClear CG-800). The pyjamas, placed in a sealed plastic package, consisted of a long-sleeved top and long pants to be worn at night for the duration of the study. Both pyjamas were made of 100% organic cotton, without dyes or preservatives, and were visually indistinguishable from each other. The *in vitro* antibacterial activity of the chitosan-coated textile was shown to persist after 30 washing cycles [16] and washing durability was studied through washing assays at 40°C [16].

### Outcomes and definitions

The primary efficacy outcome measure was mean relative and absolute change in disease severity after the intervention assessed by SCORAD [14]. The SCORAD index combines objective items reflecting disease extent, intensity and subjective items (pruritus and sleep loss) evaluated by the patient on a 10-point visual analog scale (VAS), where 0 indicates no pruritus or sleep loss and 10 indicates the worst possible pruritus and sleep loss. The total possible score ranges from 0 to 103.

Secondary outcome measures were number of patients with a minimal clinically important difference in SCORAD post-intervention; mean change in quality of life score; changes in daily pruritus and sleep loss scores; need for rescue medication; number of flares; number of totally controlled weeks (TCWs) and well-controlled weeks (WCWs); and number and severity of adverse events during the 8-week study period. Microbiological outcome measures were mean changes in colony forming units (CFUs) per 100 cm^2^ of total staphylococci (*S*. *aureus* plus coagulase negative staphylococcus species) and *S*. *aureus* isolates.

Patients were characterized according to age, gender, current medication, personal history of atopy, self-reported medical diagnosis of asthma, disease duration, and disease severity. The SCORAD index was used to classify AD as mild (score ≤15), moderate (16–39), or severe (>40) [17]. During the baseline and final visits, participants were asked to complete the Portuguese version of the Dermatology Life Quality Index (DLQI) or, if they were younger than 16 years, the children´s version of the questionnaire. Both questionnaires have been translated and validated for use in the Portuguese population [18, 19]. DLQI scores are interpreted as no effect on the patient’s life (score of 0–1), a small effect (2–5), a moderate effect (6–10), a very large effect (11–20), and an extremely large effect (21–30) [20, 21].

Participants recorded and scored daily symptoms of pruritus and sleep loss according to the 10-point VAS, and registered all medication use during the study period. Rescue medication was defined as any treatment, other than emollient, applied in response to disease worsening (i.e. escalation of treatment). A flare was defined as an episode requiring rescue medication for 3 or more consecutive days; a TCW week as a pruritus score of above 4; and a WCW as a 7-day period with need for rescue treatment or with a sleep loss or pruritus score of above 4 for no more than 2 days. [22].

### Microbiological assays

The microbiological profile was assessed by determination of viable cell numbers of total staphylococci and *S*. *aureus* in five regions: the right and left brachial crease, right and left popliteal crease, interscapular region. The regions were assessed by sampling a 25-cm^2^ area of skin with a sterile cotton swab dipped in sterile saline solution. Samples were kept refrigerated at 4°C and were processed within a maximum of 2 hours of sampling. They were decimally diluted and plated in Mannitol Salt agar (MSA; Lab Mspread plate) and Baird-Parker agar (BPA; Lab M, Lancashire, UK) using the spread plate technique. After incubation, the colonies were counted, using MSA for total staphylococcal counts and BPA for *S*. *aureus* counts, the respective Colony Forming units /100 cm^2^ were determined.

### Adverse events

Patients were asked to inform the research team of any possible adverse events that occurred during the 8-week study period. Adverse events were classified as mild if they were easily tolerated by the patient; moderate if they interrupted the individual’s usual activities; and severe if they were potentially life-threatening. The principal investigator classified adverse events as not, possibly, probably, or definitely related to treatment.

### Statistical analysis

All efficacy outcomes were analyzed using intent-to-treat populations based on the treatment group assigned at randomization.

Analysis of variance (ANOVA) supplemented by a repeated measures analysis was used for the primary outcome. A mixed effects models with random intercept and time slope by individual were used to estimate the interaction term to compare time trends between groups for number of days per week with need for rescue medication and daily symptoms. Chi-squared, Fisher exact and McNemar test were used for secondary outcomes; Wilcoxon signed rank test and Mann Whitney test for non-parametric analysis; *t* test for parametric analysis. All analyses, summaries, and listings were performed with SPSS software, version 20.0.

### Ethics

The university and hospital ethics committees approved the present study (ClinicalTrials.gov Identifier: NCT01597817). Written informed consent was obtained from each participant and from parents, caretakers, or guardians on behalf of the minors/children prior to enrolment.

The trial was performed in compliance with the Declaration of Helsinki and according to good clinical practice.

## Results

### Patients characteristics

No major imbalances were found in the baseline characteristics of the individuals included in the placebo and chitosan groups: most patients were adult, with AD for more than 10 years, more than half were female, the majority were atopic and had self reported previous history of asthma (Table 1). Oral antihistamines and topical steroids were used by most patients, almost half had been prescribed at least once oral steroids in the last year and a systemic immunosupressor such as cyclopsorin in 17% overall. Similar proportion of participants with mild (2 versus 5), moderate (19 versus 14) and severe (22 versus 16) AD occurred respectively in chitosan and placebo intervened groups.

**Table 1:** Baseline characteristics of atopic dermatitis patients by chitosan intervention group (*N* = 43) and placebo group (*N* = 35).

	Chitosan	Placebo	P-value
Age, y	23 (19–34)	26 (18–31)	0.61^§^
Female, *n* (%)	23 (53)	21 (60)	0.86*
Disease duration, y	18 (10–24)	12.0 (6–20)	0.31^§^
SCORAD (0–103)	44 (25–52)	38 (22–65)	0.72^§^
Current medication			
Antihistamines, *n* (%)	36 (84)	32 (91)	0.50*
Topical corticosteroids, *n* (%)	37 (86)	27 (77)	0.18*
Oral corticosteroids, *n* (%)	15 (35)	16 (46)	0.58*
Calcineurin inhibitors, *n* (%)	12 (28)	16 (46)	0.18*
Oral immunsupressors, *n* (%)	9 (21)	4 (11)	0.13^**ε**^
Diary scores			
Pruritus (0–10)	4 (2–4)	3 (2–5)	0.92^§^
Sleep loss (0–10)	2 (1–4)	1 (0–3)	0.31^§^
DLQI score	7 (5–12)	7 (5–12)	0.93^§^
Atopic, *n* (%)	29 (70)	21 (60)	0.29*
Asthmatic, *n* (%)	21 (49)	18 (51)	0.69*

DLQI, Dermatology Life Quality Index. Results are presented as median (interquartile range) unless stated otherwise.

^§^ Mann Whitney test.

* Chi-squared test

^**ε**^ Fisher exact test.

#### Efficacy and tolerability

**Fig 2:**
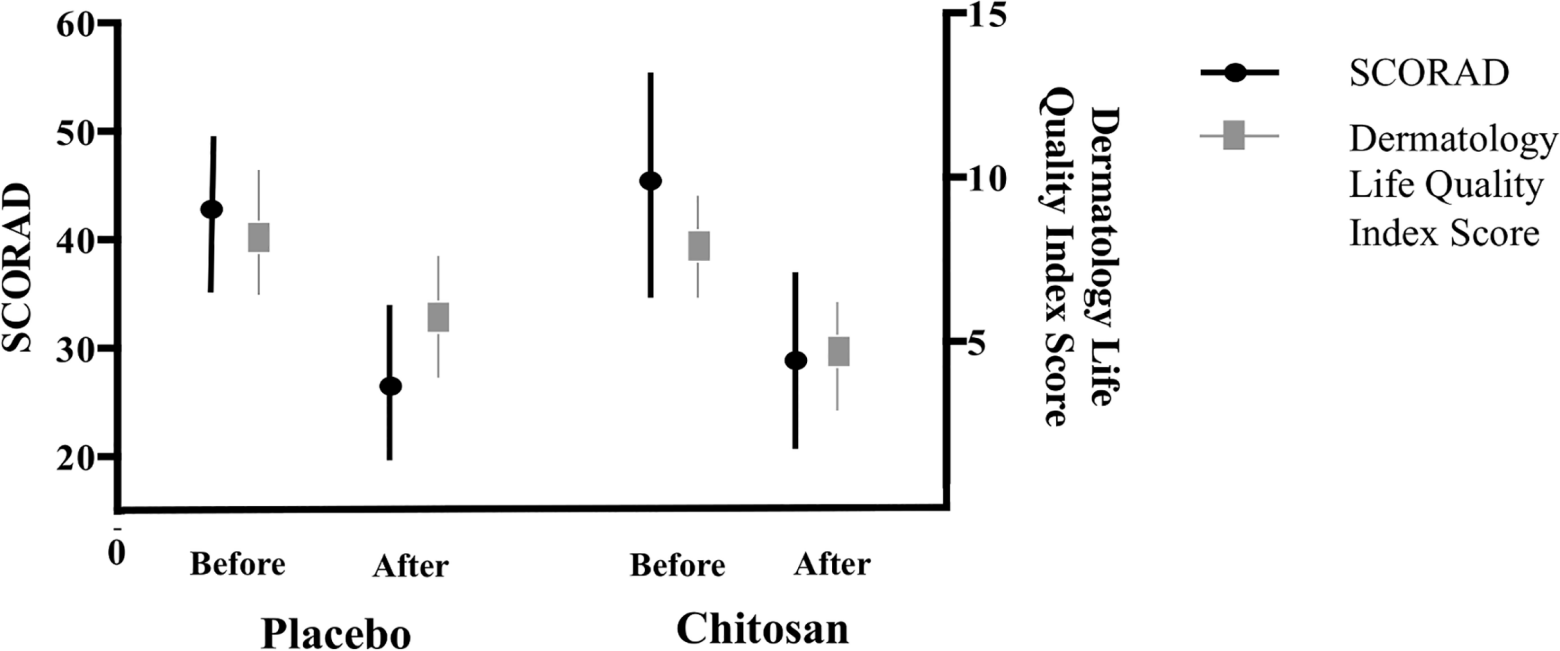
Mean SCORAD and Dermatology Life Quality Index scores (95% CI) in chitosan and placebo groups before and after intervention. CI-confidence interval.

After the 8-week intervention period there was a significant improvement in SCORAD from baseline for both the chitosan group and the placebo group (improvement of 43.8%, 95% CI: 30.9 to 55.9; *P* = 0.01 vs. 16.5%, 95% CI: -21.6 to 54.6; *P* = 0.02). The respective absolute reductions in SCORAD scores were from 44.2 (95% CI: 34.5 to 53.9) to 29.4 (95% CI: 21.4 to 37.4) and 41.4 (95% CI: 34.3 to 48.6) to 25.7 (95% CI: 18.3 to 33.1); (Fig 2). No significant differences were observed between groups for changes in SCORAD.

**Fig 3:**
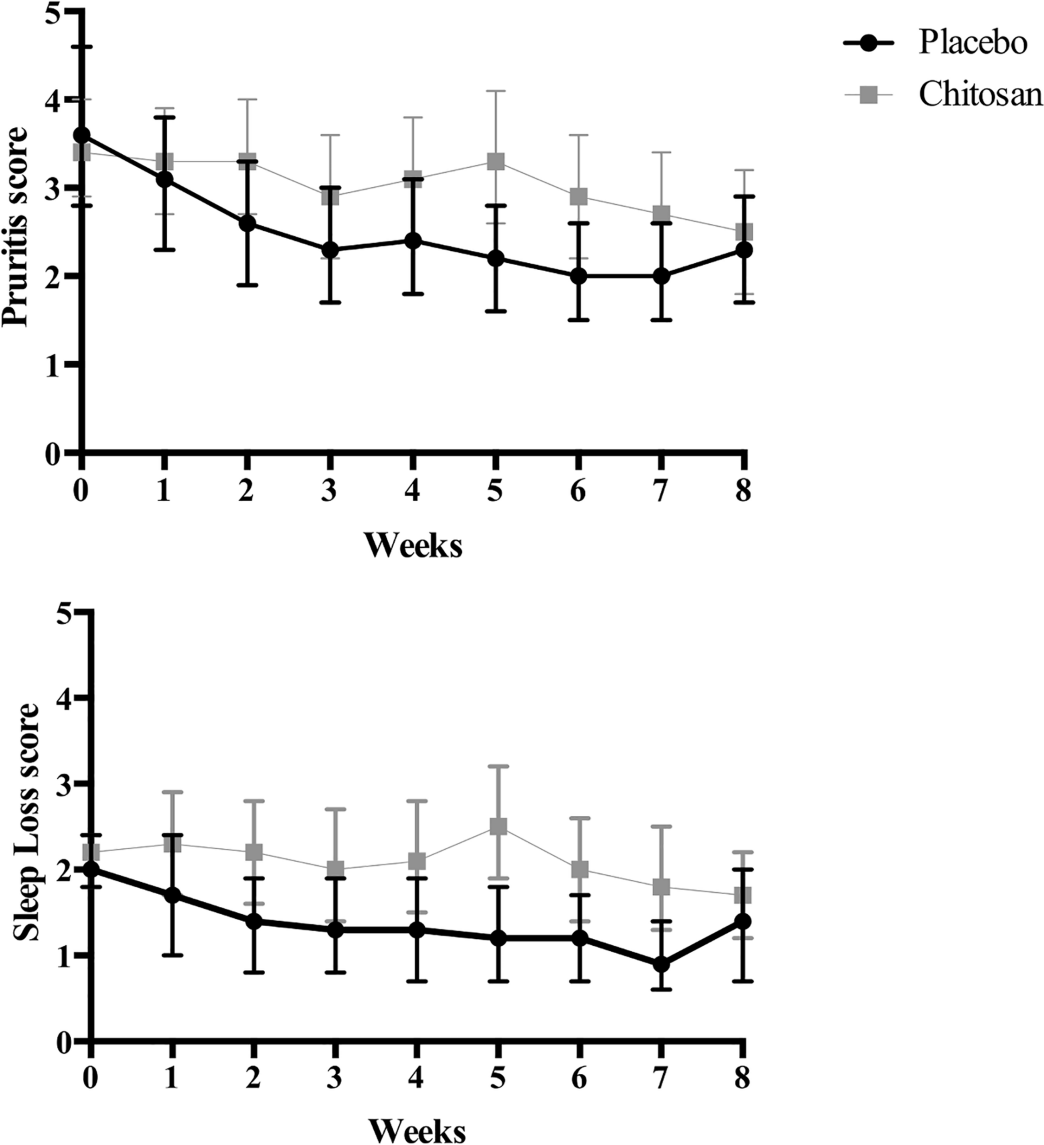
Mean (95% CI) weekly pruritus and sleep loss scores in chitosan and placebo groups throughout the intervention period. CI-confidence interval.

The improvement in DLQI scores from baseline was 36% (95% CI: 23.5 to 48.1) in the chitosan group (8.0 [9.3–6.7] to 4.8 [6.2–3.4], *P* = 0.02) and 25% (95% CI: 6.0–44.1) in the placebo group (8.3 [10.4–6.3] to 5.6 [7.7–3.5], *P* = 0.28) (Fig 2). There were no significant differences between both groups. The proportion of individuals with a clinically meaningful improvement in SCORAD was 25 (67%) in the chitosan group and 20 (63%) in the placebo group. No significant effect was observed either on daily pruritus or sleep loss scores (Fig 3 and S2 Table), need for rescue medication, or number of flares or totally controlled weeks and well controlled weeks (Table 2).

**Table 2:** Differences in efficacy outcomes in chitosan and placebo groups after intervention.

	Chitosan	Placebo	P-value for difference^§^
Rescue medication, days	2.0 (0.0–8.3)	5.0 (0.0–15.5)	0.82
Flares	0.0 (0.0–1.0)	0.0 (0.0–1.0)	0.73
Totally controlled weeks	4.0 (0.8–7.0)	4.5 (1.8–8.0)	0.43
Well controlled weeks	1.5 (0.8–3.0)	2.0 (0.0–3.0)	0.82
Uncontrolled weeks	1.0 (0.0–4.3)	1.0 (0.0–5.0)	0.94

Median (interquartile range)

^§^ Mann Whitney test.

**Fig 4:**
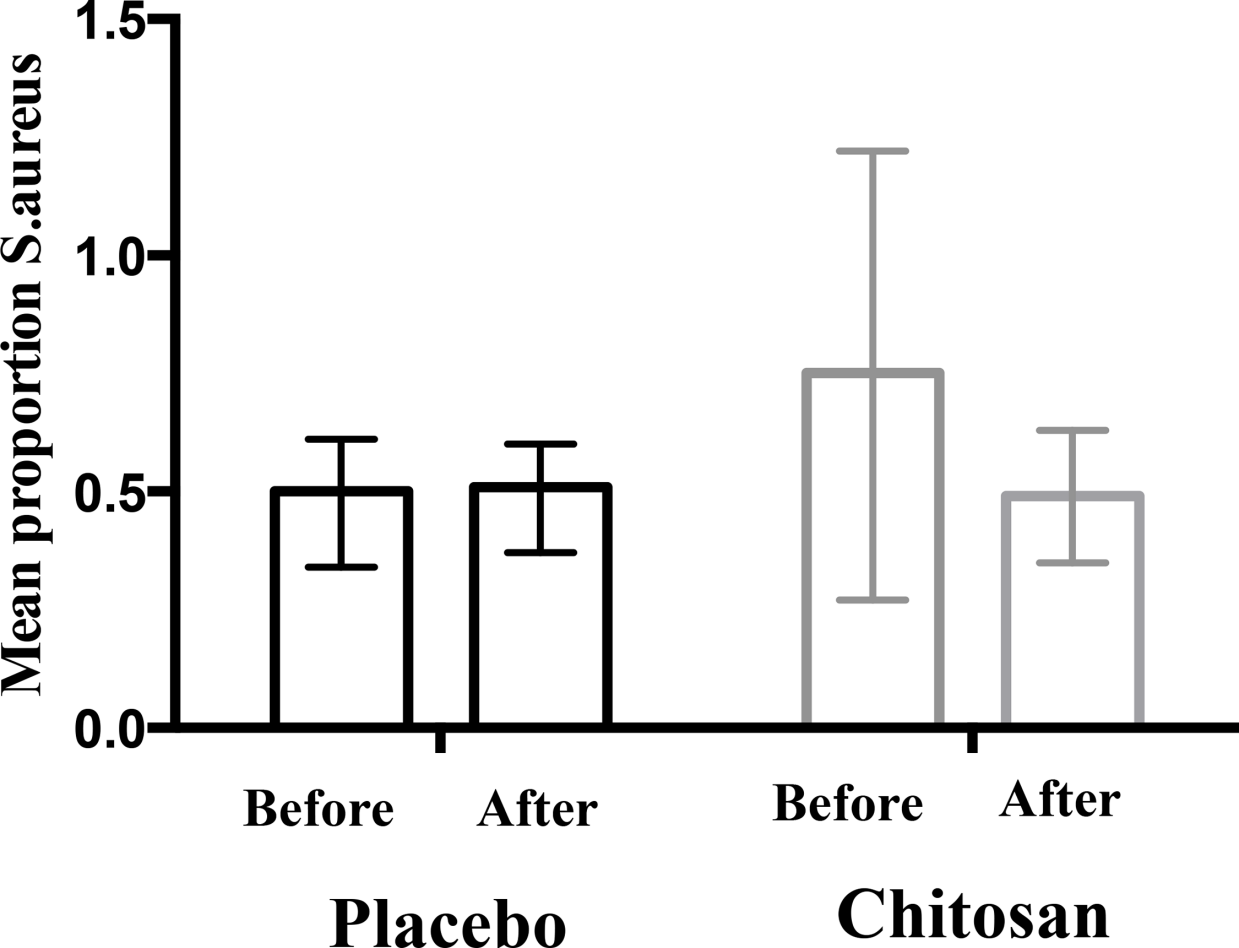
Mean (95% CI) *Staphylococcus aureus* colony forming units in all regions as proportion of total staphylococcal counts before and after intervention in placebo and chitosan groups. CI-confidence interval.

**Fig 5:**
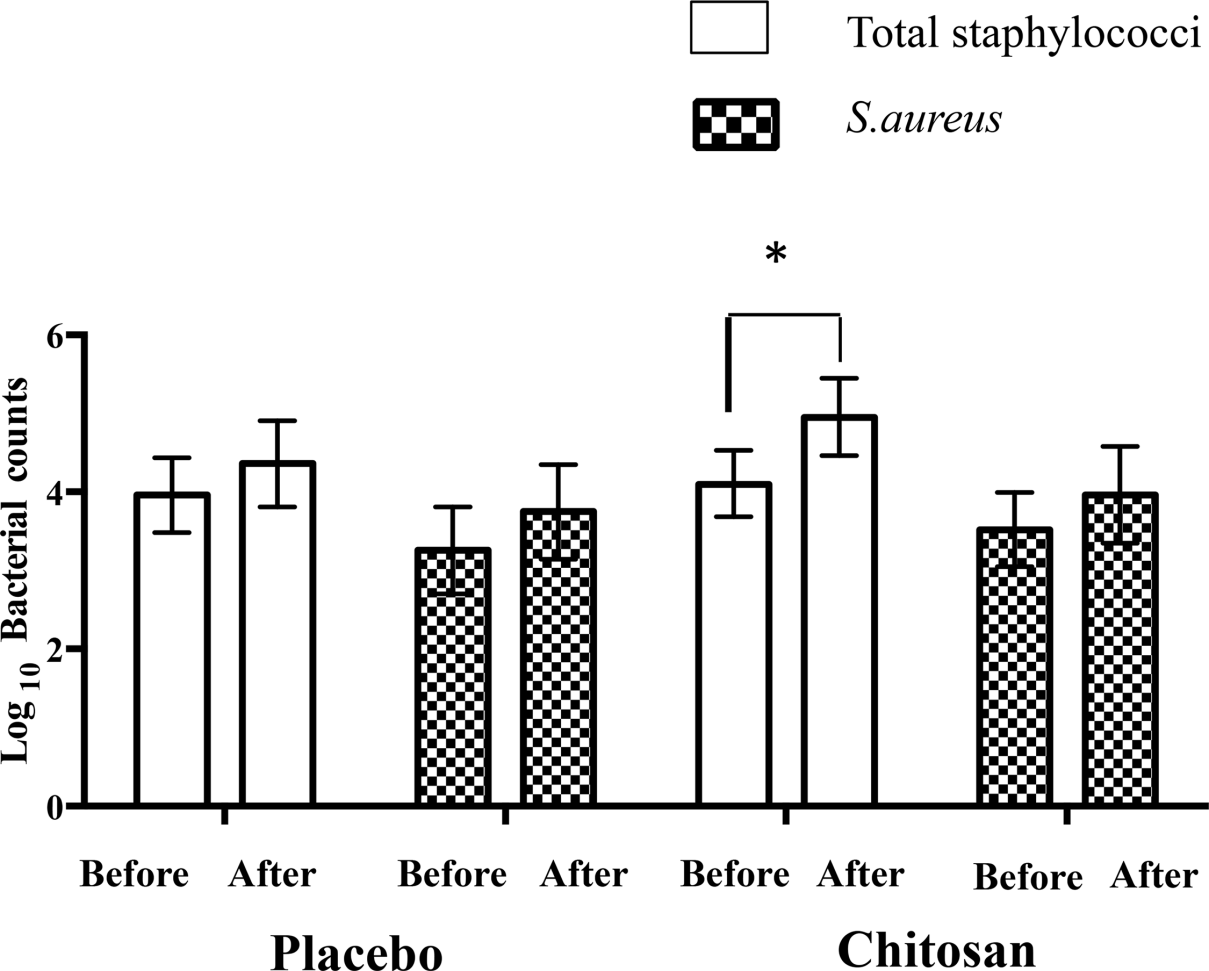
Mean (95% CI) Log10 total staphylococci and Log10 Staphylococcus aureus counts for all regions sampled in chitosan and placebo groups before and after intervention. CI-confidence interval *P = 0.01, Wilcoxon signed rank test.

Rescue medication defined as any treatment, other than emollient, that was applied in response to a worsening of the disease, corresponding to dosing up treatment; a flare as need of rescue medication for three or more consecutive days; a totally controlled week as a seven-day period without need of rescue treatment and without any days of sleep loss or pruritus score above 4; a well controlled week if rescue treatment and sleep loss or pruritus score above 4 occurred for no more than 2 days, and any other week that did not correspond to the previous definitions of totally and well controlled weeks was classified as no controlled;

Most patients had identification of Staphylococci species in at least one sampled region with no significant changes after the intervention or for changes between groups (Table 3). There a was a decrease in the percentage of patients with identification of S.aureus from 68% to 55% in chitosan group in contrast with an increase in placebo group (from 53% to 64%) that did not reach statistical significance (Table 3). The mean proportion of S.aureus counts versus total staphylococcal counts showed no significant differences after intervention for both groups on the five sample regions (right arm, left arm, right leg, left leg, neck) (Table 3) neither when considering all regions (Fig 4). When considering total bacterial counts there was a significant increase of the mean total staphylococcal count in the chitosan group (*P* = 0.02), with no other differences (Fig 5).

**Table 3:** Skin microbiological profile in chitosan and placebo groups before and after intervention.

	Chitosan			Placebo			Chitosan vs Placebo
	Before (*N* = 38)	After (*N* = 34)	*P*-value	Before (*N* = 30)	After (*N* = 28)	*P*-value	*P*-value
Staphylococci +, *n* (%) of patients	34 (85)	30 (75)	0. 71 ^P^	26 (87)	23 (82)	0.92 ^P^	0.68^**Φ**^
*S*. *aureus* +, n (%) of patients	27 (68)	22 (55)	0.92 ^P^	18 (53)	18 (64)	0.72 ^P^	0.69.^**Φ**^
% CFU *S*. *aureus*/total staphylococci							
Right arm	58 (14–74)	55 (18–68)	0.43*	71 (38–94)	81 (31–96)	0.21*	0.14^§^
Left arm	62 (12–68)	61 (12–77)	0.94*	65 (38–81)	67 (39–70)	0.42*	0.34 ^§^
Right leg	66 (18–73)	65 (13–76)	0.32*	68 (22–78)	67 (22–89)	0.52*	0.92 ^§^
Left leg	70 (18–82)	69 (25–77)	0.91*	69 (24–78)	71 (36–88)	0.83*	0.73 ^§^
Neck	58 (22–71)	42 (22–61)	0.11*	74 (21–80)	76 (29–92)	0.34*	0.93 ^§^

CFU, colony-forming units. Median (interquartile range) unless stated otherwise.

^P^ McNemar test

^**Φ**^ Chi-squared test

* Wilcoxon signed rank test

^§^ Mann Whitney test.

The chitosan-coated pyjamas were well tolerated. One patient in the chitosan group decided to withdraw at week 4 due to an AD flare, but no causal link was established.

## Discussion

In this randomized controlled trial chitosan coated textiles, used for 8 weeks, were associated with a non-significant trend of disease severity improvement. Moreover, this effect was related with a significant increase of skin coagulase negative Staphylococci.

Our study has some limitations. First, since this is a pilot study the number of participants and outcomes assessed may have been not sufficient to detect significant differences. However, based on previously published minimally clinically important differences for SCORAD, the study was designed to be sufficiently powered to detect meaningful differences. However, post hoc analysis analysing the high range of confidence limits in the control group versus the active one suggested this may have not been the case. Although we only used validated outcomes in line with recently published recommendations [23], DLQI did not fit Rasch analysis in previous studies [24] and its children´s version has not been tested till now. Nevertheless it has been previously validated in Portuguese population[19] and in our study we found an intraclass correlation coefficient in placebo group of 0.73 signifying a good reproducibility. Second, the study participants were adolescents and adults with long-standing atopic dermatitis and there would probably be a greater likelihood of detecting clinically significant improvement in adults with more severe disease. Thirdly, because no *a priori* data exist on the duration of the intervention and its *in vivo* effects, we cannot rule out that longer skin contact with chitosan may have elicited a more pronounced effect. However, the participants were instructed to wear the pyjamas every night for the duration of the study, as we wished to target a critical period. Finally, the fact that the patients were allowed to use rescue medication may have influenced the effect of the intervention. However, this was corrected for in the mixed effects model and the effect on clinical outcome analyses should therefore be minimal. This is the first trial to evaluate the utility of a biopolymer in patients with AD and, so far, it is the largest study of functional textiles. Another innovative aspect was the evaluation of other staphylococcal species than *S*.*aureus* [25].

Chitosan has exhibited skin repair potential in wounds and antimicrobial action in diverse medical fields [26–29], explaining why chitosan could potentially improve disease severity in patients prone to non-commensal bacteria colonization and skin barrier impairment. In the present study, chitosan-coated garments had no effect on the skin *S*.*aureus* counts but surprisingly, we observed in the chitosan group an increase in total staphylococci counts independently of *S*. *aureus*, corresponding to coagulase negative staphylococci species (CNS). The increase of CNS on the skin of AD patients has been already reported eliciting different explanations for this fact: some authors argue that it may be the result of a mutualistic relationship or represent a compensatory or antagonistic mechanism to control *S*.*aureus* [30]. Our data supports the hypothesis that chitosan may had exerted a specific inhibitory effect upon *S*. *aureus*, allowing the proliferation of other staphylococcal species. Nevertheless, the clinical significance of this observation is exploratory.

The observed placebo effect on disease severity may possibly be due to the improved skin comfort provided by the long-sleeved organic cotton pyjamas used, and/or to the patients’ enthusiasm about participating in a clinical trial with a new product.

The significant improvement on quality of life with chitosan treatment was probably related to reduction in AD severity in this group. Considering that sample size was calculated to detect changes in SCORAD index, we cannot exclude that more patients were needed to elicit a more pronounced effect on this outcome.

The intervention was well tolerated over the 8-week study period. There was one moderate adverse event, deemed to be unrelated to treatment, in the chitosan group. Safety of functional textiles is a controversial issue since some authors have claimed that the use of antimicrobial compounds could remove bacteria from the skin surface and pave the way for invasion by pathogenic bacteria, such as methicillin-resistant *S*. *aureus* [31].

Atopic dermatitis is a complex disease that requires a multidimensional treatment approach. The possibility of modulating the skin microbiome, namely its staphylococcal community, which has long been recognized as one of the main determinants of skin inflammation, is an appealing strategy. The use of functional textiles is also appealing because of their potential action targeting the skin surface and their favourable safety profile and convenience of use. Results from our randomized controlled trial showed that chitosan coated textiles may impact on disease severity by modulating skin staphylococcal profile. Moreover, a potential effect in quality of life may be considered.

## Supporting Information

S1 CONSORT Checklist Consort Checklist. (DOCX)

S1 PROTOCOL Ethic Committee protocol. (DOCX)

S1 Table Study schedule D, day; W, week. (DOCX)

S2 Table Mixed effects model comparing time trends between chitosan and placebo groups. SD-standard deviation.
(DOCX)
