# Supplementary material for: Chitosan Coated Textiles May Improve Atopic Dermatitis Severity by Modulating Skin Staphylococcal Profile: A Randomized Controlled Trial
Source: PLoS One. 2015 Nov 30;10(11):e0142844. doi: 10.1371/journal.pone.0142844 (PMC4664262; doi:10.1371/journal.pone.0142844)
Supplement: S1 PROTOCOL — (DOCX) [file pone.0142844.s002.docx]

**Supporting information: Ethics committee protocol**

**Biofunctional textiles in the management of atopic dermatitis**

**Investigator: Cristina Lopes**

**Working team:**

**Cristina Lopes,** Oksana Sokhatska, Carmo Palmares, José Soares, Freni Tavaria, Manuela Pintado Osvaldo.Correia, Luís Delgado,**André Moreira**

**August 2011**

1. PROTOCOL SUMMARY 3

Key Roles 5

2. Introduction: Background Information and Scientific Rationale 6

2.1 Background Information and Rationale 6

2.2 Potential Risks and Benefits 7

3. Objectives 8

3.1 Study Objectives 8

3.2 Study Outcome Measures 8

4. Study Design 10

5. Study Enrollment and Withdrawal 11

5.1 Subject Inclusion Criteria 11

5.2 Subject Exclusion Criteria 11

5.3 Strategies for Recruitment and Retention 11

5.4 Treatment Assignment Procedures 12

6. Study Intervention/Investigational Product 13

6.1 Study Product Description 13

6.2 Dosage, Preparation and Administration of Study Intervention/Investigational Product 13

6.3 Accountability Procedures for the Study Intervention/Investigational Product(s) 13

6.4 Concomitant Medications/Treatments 13

7. Study Schedule 15

8. Statistical Considerations 17

8.1 Study Hypotheses 17

8.2 Sample Size Considerations 17

9. Source Documents and Access to Source Data/Documents 18

10. Ethics/Protection of Human Subjects 19

10.1 Ethical Standard 19

10.2 Institutional Review Board 19

10. Informed Consent Process 19

10.4. Exclusion of Special Populations 19

10.5 Subject Confidentiality 20

10.6 Study Discontinuation 20

11. Data Handling and Record Keeping 21

11.1 Data Management Responsibilities 21

11.2 Data Capture Methods 21

11.3 Types of Data 21

12. Publication Policy 22

13. Planning: 23

13.1 Chronogram 23

13.2 Human Resources: 23

14. Funding 25

15. Literature References 26

## 1. PROTOCOL SUMMARY

| **Title:** | Biofunctional textiles in the management of atopic dermatitis |
| --- | --- |
| **Précis:** | Atopic dermatitis (AD) is a chronic inflammatory skin disease characterized by exacerbations and remission of intensely pruritic lesions of variable location. AD may be acute (short-term and severe) with predominantly redness, vesicles and oozing, or it may be chronic (long-term) with scaling, skin thickening, altered pigmentation and exaggerated surface markings. The condition affects mainly the creases of the elbows and knees, and the face and neck, although it can affect any part of the body. The severity of AD is variable, ranging from localized mild scaling to generalized involvement of the whole body. Itching is the predominant symptom which can induce a vicious cycle of scratching, leading to skin damage. There is a tendency to lifelong dry sensitive skin. Skin of AD is often colonized by Staphylococcus aureus contributing to perpetuating cutaneous inflammation. AD treatment is based on skin hydration, identification and elimination of flare factors, and pharmacologic therapy. Biofunctional textiles are emerging as new and complementary tools . Chitosan is a natural polysaccharide with in vitro anti-microbial activity and regenerating properties. We aim to evaluate the effect of a textile coated with chitosan in management of AD as well as its impact on systemic inflammation and skin flora namely on colonization by Staphylococcus aureus .  Patients with medical diagnosis of AD are selected. Clinical data are collected and written information is provided. After enrollment patients are randomized to active group (cotton textiles impregnated with chitosan) or control group (cotton textiles). Patients are asked to use a Tshirt and pants as pijamas for a period of 8 weeks. Outcomes are clinical improvement measured by SCORAD (severity score of AD including subjective symptoms as sleep disturbance and pruritus and intensity and severity of skin lesions), quality of life, inflammatory serum markers and skin microbiological assays. |
| **Objectives:** | To assess the efficacy of a natural biopolymer textile in the management of atopic dermatitis, and to characterize its immunomodulatory properties and impact on skin microbiological flora . |
| **Setting:** | Immunology Department, Medical Faculty , Porto University |
| **Subjects:** | Portuguese patients, of any gender and any race, aged over 12 years old; |
| **Study design:** | Randomized, double-blind, parallel placebo-controlled study |
| **Estimated time to complete enrollment:** | 6 months |

## Key Roles

| **Individuals:** | **Principal Investigator:**  **Cristina Lopes,** MD,Immunology Department, Faculty of Medicine, University of Porto, Allergy Unit , Hospital Pedro Hispano, Matosinhos |
| --- | --- |
|  | **Working team:**  **Oksana Sokhatska**, Technical assistant, Immunology Department Faculty of Medicine, University of Porto  **Carmo Palmares**, Main Assistant, Immunology Department, Faculty of Medicine, University of Porto  **José Soares**, Microbiologist, Biotecnhology School , Catolic University, Porto  **Freni Tavaria**, PhD, Biotechnology School , Catolic University, Porto  **Manuela Pintado,** PhD, Biotecnhology School , Catolic University, Porto  **Osvaldo Correia,** MD, PhD, Faculty of Medicine, University of Porto, Epidermis, Instituto Cuf Porto  **Luis** **Delgado**, MD, PhD, Faculty of Medicine, University of Porto, Hospital S.João Porto  **André Moreira**, MD, PhD, Faculty of Medicine, University of Porto, Hospital S.João Porto |
|  |  |
| **Institutions:** | Faculty of Medicine, University of Porto  Alameda Prof. Hernâni Monteiro  4200 – 319 Porto, Portugal |

## 2. Introduction: Background Information and Scientific Rationale

### 2.1 Background Information and Rationale

Atopic dermatitis (AD) is a chronic inflammatory skin disease characterized by exacerbations and remission of intensely pruritic lesions of variable location. It affects predominantly children but when persisting in adolescence and adulthood tends to be more severe (1). Studies in the first half of the twentieth century have shown an incidence of 2%–3% (2), while more recent surveys have shown an increase to 9%–12% in childhood (3). Its prevalence has been found unevenly distributed over the world and in Portugal, eczema prevalence was found to be 9.3 % and 5.2% on 6-7 and 13-14 years old respectively (4).

Atopic dermatitis has a significant impact on the quality-of-life of patients and their families, comparable to other chronic diseases, and its economic impact is high (5). More than 50% of patients with AD develop asthma and other atopic disorders, adding further impact in the health and economic burden of this disease.

Its pathogenesis is not fully understood and the interaction of environment in genetic predisposed individuals seems to be determinant(4) .Besides the involvement of adaptive immunes response trough mechanisms related to IgE synthesis, structural changes of the epidermis and innate immunity dysfunction play a significant role. Patients with fillagrin (a key protein of epidermal differentiation) gene mutation, comprise not only an earlier onset of disease as well as more severe and persistent AD (5, 6). AD patients also have an increased risk of infection with certain types of microorganisms (7) that has been related to immune deficiency of innate immune system on a already disrupted skin barrier.

AD may be acute (short-term and severe) with predominantly redness, vesicles and oozing, or it may be chronic (long-term) with scaling, skin thickening, altered pigmentation and exaggerated surface markings. The condition affects mainly the creases of the elbows and knees, and the face and neck, although it can affect any part of the body. The severity of AD is variable, ranging from localized mild scaling to generalized involvement of the whole body, with redness, oozing and secondary infection. Itching is the predominant symptom which can induce a vicious cycle of scratching, leading to skin damage which in turn leads to more itching - the so called “itch scratch itch” cycle. There is a tendency to lifelong dry sensitive skin.

There is currently no cure for AD however, a wide range of treatments are employed to control symptoms (9). Emollients and topical corticosteroids are universally recommended (10) and anti-histamines, topical tacrolimus and pimecrolimus are part of pharmacological options. Probiotics, and allergen immunotherapy are used as immunomodulators with controversial effectiveness

(11). Special textiles that can contribute to preserve skin integrity, prevent loss of water and exert immunomodulatory effects are being developed as complementary tools.

The concept of “biofunctional textiles” refers to the potential ability of certain textiles to modulate skin dryness, flora and/or immune status if they are used long enough and in close contact with skin. With the development of nanotechnology it became possible to impregnate textiles with bioactive compounds. Chitosan, a natural biopolymer has antioxidant, antibacterial and antifungal properties enhancing skin repair (8).

The clinical utility of textiles impregnated with chitosan in patients with AD has never been tested.

### 2.2 Potential Risks and Benefits

##### 2.2.1 Potential Risks

Chitosan is a linear polysaccharide derived from chitin. Chitin is the second most abundant natural polymer obtained from the exoskeleton of crustaceans. It has high biocompatibility and biodegradability with various applications in the pharmaceutical industry, which includes wound dressings, gauzes, and medical sutures and also in regenerative medicine. We do not expect to have adverse events related to the use of textiles coated with chitosan. Textiles will be made of smooth cotton fiber with appropriated manufacture for patients with skin diseases (no dyes, no preservatives with sensitization potential).

##### 2.2.2. Known Potential Benefits

Chitosan possesses various biological activities (antioxidant, wound-healing accelerator, antibacterial and antifungal). It has also been investigated as scaffold for skin regeneration due to its biocompatibility, biodegradability, and bioactivity.

## 3. Objectives

### 3.1 Study Objectives

To assess the efficacy of a natural biopolymer textile in the management of atopic dermatitis

### 3.2 Study Outcome Measures

##### 3.2.1 Primary Outcome

- Investigator rated eczema severity

Clinical improvement measured by local SCORAD (score of severity of AD- supplement 1) (initial versus final, % of change).

SCORAD is composed of three different domains (A= extension B= intensity C = subjective symptoms). To determine extent, the sites affected by eczema are shaded on a drawing of a body. The rule of 9 is used to calculate the affected area (A) as a percentage of the whole body: Head and neck 9% Upper limbs 9% each , Lower limbs 18% each , Anterior trunk 18% ,Back 18% 1% each for genitals, each palm and the back of each hand. The score for each area is added up. The total area is 'A', which has a possible maximum of 100%.

A representative area of eczema is selected. In this area, the intensity of each of the following signs is assessed as none (0), mild (1), moderate (2) or severe (3).:Redness ,Swelling ,Oozing / crusting Scratch marks , Skin thickening (lichenification),Dryness (this is assessed in an area where there is no inflammation) .The intensity scores are added together to give 'B' (maximum 18).

Subjective symptoms i.e., itch and sleeplessness, are each scored by the patient or relative using a visual analogue scale where 0 is no itch (or no sleeplessness) and 10 is the worst imaginable itch (or sleeplessness). These scores are added to give 'C' (maximum 20).

- Changes in Quality of life.

Patients are asked to answer the Portuguese version of the Dermatology Life Quality Index (> 16 years old) or the children´s Dermatology Quality of Life Index (4-16 years old) at the beginning and end of the study (supplement 2 and 3)

##### 3.2.2 Secondary Outcomes

- Changes in participant rated symptoms of eczema

Patients are asked to record the severity scores of itchiness and sleep disturbance of the previous day in a diary card (10 point scale from 0-none to 10-extreme) (supplement 4)

- Changes in the need of eczema treatment

Patients are asked to record the use of topical steroids, antihistamines, oral steroids or immunosuppressive drugs on a diary card. (supplement 4)

- Immunological serum markers:

Changes in serum total IgE, specific IgE to enterotoxin A,B, C and TSST (staphylococcus enterotoxins) serum eosinophil cationic protein (ECP), blood eosinophils, C reactive protein. Changes in cytokine serum levels (RANTES, IL-31, IL-18,IL-16).

- Changes in skin microflora

Characterize the skin microflora of determined areas and determine the changes in number of colony forming units of *Staphylococcus aureus* from the beginning to the end of study.

## 4. Study Design

Double blind, placebo-controlled, parallel randomized study enrolling a sample of patients with medical diagnosis of AD.

The approximate time to complete study enrollment is about 6 months. The expected duration of subject participation is eight weeks, approximately, with one screening visit and two visits after enrollment, one at the beginning and the other at end of the study. During the eight weeks trial the patient will be asked to use cotton Tshirt and cotton pants as pijama during the night.

Visits are performed at an appropriate medical setting at Instituto Cuf, Porto. Each visit lasts approximately 45 min including medical examination, filling of one questionnaire, serum sampling and a superficial skin swab of determined areas.

## 5. Study Enrollment and Withdrawal

### 5.1 Subject Inclusion Criteria

In order to be eligible to participate in this study, a subject must meet all of the following criteria:

- Medical diagnosis of atopic dermatitis
- Age superior to three years old

### 5.2 Subject Exclusion Criteria

A potential subject who meets any of the following criteria will be excluded from participation in this study if:

- Presence of any significant illness that could interfere with the study, particularly if accompanied by immunological skin abnormalities as psoriasis
- Contact eczema, hand eczema , nummular eczema, other forms of eczema that not atopic eczema
- Anything that, in the opinion of the investigator, preclude the subject’s full compliance with or completion of the study.

### 5.3 Strategies for Recruitment and Retention

Patients who attend any Allergy, Dermatology, Pediatrics, General Practitioner consults from the North region of Portugal and have the medical diagnosis of AD are invited to participate in the trial and contact the Immunology Department of Medical Faculty of Medicine. A screening visit is scheduled.

Advertisement trough media (television and newspapers ) invites patients from the general population with medical diagnosis of atopic dermatitis to contact the Immunology Department of Medical Faculty of Medicine and schedule a screening visit.

### 5.4 Treatment Assignment Procedures

##### 5.4.1 Randomization Procedures

##### Computer generated sequence

##### 5.4.2 Masking Procedures

Patients and investigator are provided with cotton t-shirts and pants coated and non coated with chitosan. Clothes are visual indistinguishable from each other and both patients and investigators are blinded to the intervention.

##### 5.4.3 Reasons for Withdrawal

A study subject will be discontinued from participation in the study if:

- Any clinical adverse event (AE), or other medical condition or situation occurs such that continued participation in the study would not be in the best interest of the subject.
- The subject meets any exclusion criteria (either newly developed or not previously recognized).
- Subjects are free to withdraw from participation in the study at any time upon request.

##### 5.4.5 Termination of Study

The study ends when the sample size is achieved (approximately 90 patients)

This study may be prematurely terminated if, in the opinion of the investigators, there is sufficient reasonable cause.

Circumstances that may warrant termination include, but are not limited to:

- Determination of unexpected, significant, or unacceptable risk to subjects.
- Insufficient adherence to protocol requirements.
- Data are not sufficiently complete and/or evaluable.

## 6. Study Intervention/Investigational Product

### 6.1 Study Product Description

##### 6.1.1 Acquisition

Textiles will be provided by textile enterprise Crispim e Abreu Lda.

##### 6.1.2 Formulation, Packaging, and Labeling

Formulation, packaging, and labeling will be done as usual in textiles. No specific changes for the study protocol are required.

##### 6.1.3 Product distribution

Crispim e Abreu, Lda will assure storage and shipment of the material in adequate conditions to Immunology Department of Medical Faculty of Medicine.

### 6.2 Dosage, Preparation and Administration of Study Intervention/Investigational Product

Textile use will be performed according with the protocol .

### 6.3 Accountability Procedures for the Study Intervention/Investigational Product(s)

Textiles coated with chitosan will be distributed as frequent necessary for good progression of the study by Crispim e Abreu, Lda.

### 6.4 Concomitant Medications/Treatments

Patients are asked to maintain the

## 7. Study Schedule

Visit 0:

- Explain study protocol
- Review medical history to determine eligibility based on inclusion/exclusion criteria.
- Schedule visit 1

Visit 1 :

- Provide written information about the study
- Obtain signature of potential subject on written informed consent
- Perform medical examination and register SCORAD index
- Answer a quality of life questionnaire
- Patients are explained and given a diary symptoms card
- Perform skin swab of determined areas
- Perform serum sampling
- Patients are explained trial and are given a TShirt and pants
- Surveillance and medical care, if required

Visit 2 :

- Review medical history including medications history
- Perform medical examination and register SCORAD index
- Answer a quality of life questionnaire
- Deliver the diary symptoms card
- Perform skin swab of determined areas
- Perform serum sampling

## 8. Statistical Considerations

### 8.1 Study Hypotheses

The use of a textile coated with a natural biopolymer will improve clinical outcomes on patients with atopic dermatitis diminishing inflammatory biomarkers as well as the number of skin colonizing Staplylococcus aureus and expression of its virulence factors.

### 8.2 Sample Size Considerations

Considering a parallel trial with a significant level of 0.05 and statiscal power of 0.8 based on difference of means and standard deviation of outcome SCORAD the sample size calculated was 90 patients

## 9. Source Documents and Access to Source Data/Documents

The investigator will maintain appropriate medical and research records for this trial for the purposes of quality assurance reviews, audits, and evaluation of the study progress.

## 10. Ethics/Protection of Human Subjects

### 10.1 Ethical Standard

The investigator will ensure that this study is conducted in full conformity with the principles set forth in the Declaration of Helsinki.

### 10.2 Institutional Review Board

The protocol will be submitted for approval request by the Ethical Commission.

### 10. Informed Consent Process

Consent forms will be approved and the subject will be asked to read and review the document. Upon reviewing the document, the investigator will explain the research study to the subject and answer any questions that may arise. The subject will sign the informed consent document prior to any procedures being done specifically for the study. The subjects will have the opportunity to discuss the study with their surrogates or think about it prior to agreeing to participate. The subjects may withdraw consent at any time throughout the course of the trial. A copy of the informed consent document will be given to the subjects for their records. The rights and welfare of the subjects will be protected by emphasizing to them that the quality of their medical care will not be adversely affected if they decline to participate in this study. (Supplement 6)

##### 10.3.1 Informed Consent/Assent Process (in Case of a Minor)

Extensive discussion of risks and possible benefits of this procedure will be provided to the parents or subject’s legally authorized representative. Consent forms describing in detail the study procedures and risks are given to parents or subject’s legally authorized representative and written documentation of informed consent is required prior to starting intervention. Parents or subject’s legally authorized representative will be asked to read and review the document and sign prior to any procedure being done specifically for the study. (Supplement 7)

### 10.4. Exclusion of Special Populations

Children under twelve years-old .

### 10.5 Subject Confidentiality

Subject confidentiality is strictly held in trust by the participating investigators. This confidentiality is extended to the clinical information relating to participating subjects.

The study protocol, documentation, data, and all other information generated will be held in strict confidence. No information concerning the study or the data will be released to any unauthorized third party.

### 10.6 Study Discontinuation

In the event that the study is discontinued, patients will not be affected.

## 11. Data Handling and Record Keeping

The investigator is responsible to ensure the accuracy, completeness, legibility, and timeliness of the data reported. All source documents should be completed in a neat, legible manner to ensure accurate interpretation of data. Dark ink is required to ensure clarity of reproduced copies.

Copies of the electronic CRF (eCRF) will be provided for use as source documents and maintained for recording data for each subject enrolled in the study. Data reported in the eCRF derived from source documents should be consistent with the source documents or the discrepancies should be explained

### 11.1 Data Management Responsibilities

All source documents and laboratory reports must be reviewed by the clinical team and data entry staff, who will ensure that they are accurate and complete. Adverse events must be graded, assessed for severity and causality, and reviewed by the site investigator.

Data collection is the responsibility of the clinical trial staff. During the study, the investigator must maintain complete and accurate documentation for the study.

### 11.2 Data Capture Methods

Clinical data (including concomitant medications, and expected adverse reactions data) and clinical laboratory data will be entered into a data capture system. The data system includes password protection and internal quality checks, such as automatic range checks, to identify data that appear inconsistent, incomplete, or inaccurate. Clinical data will be entered directly from the source documents.

### 11.3 Types of Data

Data for this study will include age, gender, clinical data obtained by medical examination, and outcome measures – serum biomarkers, skin microbiological evaluation, quality of life data.

## 12. Publication Policy

Following completion of the study, the investigator is expected to publish the results of this research in a scientific journal.

###

## 13. Planning:

### 13.1 Chronogram

The Project has 2 years duration divided in four phases: planning, execution, analysis, conclusion

| **Phase** | **Date** | **Tasks** |
| --- | --- | --- |
| Planning/preparation | June –October 11 |  |
| Execution | November 2011-June 2012 | Patients inclusion |
| Analysis | July 2012-December 2012 | Data analysis |
| Conclusion | December 2012-June 2013 | Preparation and Manuscript submisson |

### 13.2 Human Resources:

| **Professionals** | **Academic degree** | **Specific Tasks** |
| --- | --- | --- |
| Cristina Lopes  **Principal Investigator** | *MD,* Immunology *Department,* Faculty of Medicine, University of Porto, Hospital Pedro Hispano, Matosinhos | Clinical evaluation, Data analysis |
| Carmo Palmares, | Main Assistant, Immunology Department, Faculty *of Medicine, University of Porto* | Immunological characterization |
| Oksana Sokhatska, | Technical assistant, Immunology Department *Faculty of Medicine, University of Porto* | Immunological characterization |
| José Soares | Microbiologist, Biotechnology School , Catolic University, Porto | Microbiological characterization |
| Freni Tavaria | PhD, Biotechnology School , Catolic University, Porto | Microbiological characterization |
| Manuela Pintado | PhD, Biotechnology School , Catolic University, Porto | Microbiology consultant |
| André Moreira, | MD, PhD, Faculty of Medicine, University of Porto, Hospital S.João Porto | Supervisor |
| Osvaldo Correia | MD, PhD, Epidermis, Instituto Cuf, Porto, *Faculty of Medicine, University of Porto* | Dermatologist Consultant |
| Luis Delgado | MD, PhD, Faculty of Medicine, University of Porto, Hospital S.João Porto | Immunology Consultant |

## 14. Funding

Funding supported by Serviço e Laboratório de Imunologia, Faculdade de Medicina da Universidade do Porto

## 15. Literature References

1. Eigenmann PA. Clinical features and diagnostic criteria of atopic dermatitis in relation to age. Pediatr Allergy Immunol. 2001;12 Suppl 14:69-74.

2. WC. S. The incidence of major allergic diseases in Colorado Springs. JAMA 1939 1939;112:2034–7.

3. Taylor B, Wadsworth J, Wadsworth M, Peckham C. Changes in the reported prevalence of childhood eczema since the 1939-45 war. Lancet. 1984 Dec 1;2(8414):1255-7.

4. Odhiambo JA, Williams HC, Clayton TO, Robertson CF, Asher MI. Global variations in prevalence of eczema symptoms in children from ISAAC Phase Three. J Allergy Clin Immunol. 2009 Dec;124(6):1251-8 e23.

5. Akdis CA, Akdis M, Bieber T, Bindslev-Jensen C, Boguniewicz M, Eigenmann P, et al. Diagnosis and treatment of atopic dermatitis in children and adults: European Academy of Allergology and Clinical Immunology/American Academy of Allergy, Asthma and Immunology/PRACTALL Consensus Report. Allergy. 2006 Aug;61(8):969-87.

6. Gauger A, Mempel M, Schekatz A, Schafer T, Ring J, Abeck D. Silver-coated textiles reduce Staphylococcus aureus colonization in patients with atopic eczema. Dermatology. 2003;207(1):15-21.

7. Gauger A, Fischer S, Mempel M, Schaefer T, Foelster-Holst R, Abeck D, et al. Efficacy and functionality of silver-coated textiles in patients with atopic eczema. J Eur Acad Dermatol Venereol. 2006 May;20(5):534-41.

8. Uchida Y, M. Izume, and A. Ohtakara. Preparation of chitosan oligomers with purified chitosanase and its application,. 1989:373-82.

9. Thomas Jung, Georg Stingl, Atopic dermatitis: Therapeutic concepts evolving from new

pathophysiologic insights. J Allergy Clin Immunol 2008

10. [Boguniewicz M](http://www.ncbi.nlm.nih.gov/pubmed?term=%22Boguniewicz%20M%22%5BAuthor%5D)*,* [Leung DY](http://www.ncbi.nlm.nih.gov/pubmed?term=%22Leung%20DY%22%5BAuthor%5D)*..; Recent insights into atopic dermatitis and implications for management of infectious complications.* [J Allergy Clin Immunol.](http://www.ncbi.nlm.nih.gov/pubmed/20109729) *2010 Jan;125(1):4-13*

11. [Bussmann C](http://www.ncbi.nlm.nih.gov/pubmed?term=%22Bussmann%20C%22%5BAuthor%5D)*,* [Böckenhoff A](http://www.ncbi.nlm.nih.gov/pubmed?term=%22B%C3%B6ckenhoff%20A%22%5BAuthor%5D)*,* [Henke H](http://www.ncbi.nlm.nih.gov/pubmed?term=%22Henke%20H%22%5BAuthor%5D)*,* [Werfel T](http://www.ncbi.nlm.nih.gov/pubmed?term=%22Werfel%20T%22%5BAuthor%5D)*,* [Novak N](http://www.ncbi.nlm.nih.gov/pubmed?term=%22Novak%20N%22%5BAuthor%5D) *Does allergen-specific immunotherapy represent a therapeutic option for patients with atopic dermatitis?*

[J Allergy Clin Immunol.](http://www.ncbi.nlm.nih.gov/pubmed/17157659) *2006 Dec;118(6):1292-8*.
