# Supplementary material for: Chitosan Coated Textiles May Improve Atopic Dermatitis Severity by Modulating Skin Staphylococcal Profile: A Randomized Controlled Trial
Source: PLoS One. 2015 Nov 30;10(11):e0142844. doi: 10.1371/journal.pone.0142844 (PMC4664262; doi:10.1371/journal.pone.0142844)
Supplement: S1 Table — (DOCX) [file pone.0142844.s003.docx]

|  | Screening | Baseline | Daily records | End |
| --- | --- | --- | --- | --- |
|  | (W -2) | (D 0) | W 0 to 8 | (W 8) |
| Informed consent | √ |  |  |  |
| Demographic characteristics | √ |  |  |  |
| Inclusion/exclusion criteria check | √ | √ | √ | √ |
| Randomization |  | √ |  |  |
| SCORAD index | √ | √ |  | √ |
| Dermatology Life Quality Index |  | √ |  | √ |
| Skin microbiological characterization |  | √ |  | √ |
| Daily pruritus score |  | √ | √ |  |
| Daily sleep loss score |  |  | √ |  |
| Rescue medication |  |  | √ |  |
| Current medication | √ |  | √ |  |
| Adverse event |  |  | √ | √ |

D, day; W, week.
