# Supplementary material for: Chitosan Coated Textiles May Improve Atopic Dermatitis Severity by Modulating Skin Staphylococcal Profile: A Randomized Controlled Trial
Source: PLoS One. 2015 Nov 30;10(11):e0142844. doi: 10.1371/journal.pone.0142844 (PMC4664262; doi:10.1371/journal.pone.0142844)
Supplement: S2 Table — SD-standard deviation. (DOCX) [file pone.0142844.s004.docx]

|  | Beta | p |
| --- | --- | --- |
| Sleep loss symptoms |  |  |
| Fixed effects |  |  |
| Control (1) | Ref. |  |
| Intervention (2) | 0.723 | 0.086 |
| Time (week) | -0.059 | 0.074 |
| Time x Intervention (2) | -0.001 | 0.99 |
| Random Effects |  |  |
| SD (Intercept) | 1.54 |  |
| SD (time) | 0.13 |  |
| Pruritus |  |  |
| Fixed effects |  |  |
| Control (1) | Ref. |  |
| Intervention(2) | 0.506 | 0.23 |
| Time (week) | -0.111 | 0.001 |
| Time x Intervention (2) | 0.014 | 0.76 |
| Random Effects |  |  |
| Sd (Intercept) | 1.536 |  |
| Sd (time) | 0.122 |  |
| Rescue medication |  |  |
| Fixed effect |  |  |
| Control (1) | Ref. |  |
| Intervention (2) | -0.407 | 0.23 |
| Time (week) | -0.073 | 0.05 |
| Time x Intervention(2) | 0.011 | 0.84 |
| Random Effects |  |  |
| Sd (Intercept) | 1.11 |  |
| Sd (time) | 0.11 |  |
